# A Biological-Driven Approach to Explore Dose-Escalated Ultra-Hypofractionation in Breast Cancer Radiotherapy

**DOI:** 10.3390/biomedicines13092154

**Published:** 2025-09-04

**Authors:** Marco Calvaruso, Denis Panizza, Riccardo Ray Colciago, Valeria Faccenda, Gaia Pucci, Elena De Ponti, Giusi Irma Forte, Giorgio Russo, Luigi Minafra, Stefano Arcangeli

**Affiliations:** 1 Institute of Bioimaging and Complex Biological Systems—National Reasearch Council (IBSBC—CNR), 90015 Cefalù, Italy; marco.calvaruso@cnr.it (M.C.); gaiapucci@cnr.it (G.P.); giusiirma.forte@cnr.it (G.I.F.); giorgio-russo@cnr.it (G.R.); luigi.minafra@cnr.it (L.M.); 2 Medical Physics, Fondazione IRCCS San Gerardo Dei Tintori, 20900 Monza, Italy; denis.panizza@irccs-sangerardo.it (D.P.); valeria.faccenda@irccs-sangerardo.it (V.F.); elena.deponti@irccs-sangerardo.it (E.D.P.); 3 Medicine and Surgery Department, University of Milan Bicocca, 20900 Milano, Italy; stefano.arcangeli@unimib.it; 4 Radiation Oncology, Fondazione IRCCS San Gerardo Dei Tintori, 20900 Monza, Italy

**Keywords:** triple-negative breast cancer, radiobiology, ultra-hypofractionation, dosimetry

## Abstract

To explore a more personalized approach to radiation therapy for adjuvant whole-breast irradiation in triple-negative breast cancer (TNBC), we analyzed the cell lines BT549 and MDA-MB-231 as in vitro models for radiobiological characterization. The local disease-free survival (LSR) values were determined for both cell lines’ median, maximum, and minimum α and β parameters to achieve an LSR probability of close to 100% in a five-fraction schedule. Based on these findings, fifteen treatment plans were created for BC to simulate the proposed dose schedule. For the MDA-MB-231 cell line, the α/β ratios were 3.79 Gy (minimum), 15 Gy (maximum), and 7 Gy (median). For the BT-549 cell line, the α/β ratios were 5.95 Gy (minimum), 22.93 Gy (maximum), and 16.51 Gy (median). To achieve an LSR probability of close to 100%, the required doses per fraction were 5.2 Gy, 5.3 Gy, and 7.3 Gy for MDA-MB-231 and 8 Gy, 9.1 Gy, and 9.9 Gy for BT-549. We selected the highest dose per fraction, 9.9 Gy × 5, to simulate the worst-case scenario. To achieve 100% cell death effectiveness in TNBC, it is likely that higher radiation doses are required—doses that are not feasible within the setting of adjuvant whole-breast irradiation. Our model, which relies on the intrinsic biological features of the tumor, paves the way to reach more tailored RT plans and to improve the classic LQ model.

## 1. Introduction

Radiotherapy (RT) represents one of the three pillars in cancer treatment, together with chemotherapy and surgery. In the latest years, conventional RT has evolved from the technical point of view. Modern techniques such as intensity-modulated radiotherapy (IMRT), image-guided radiation therapy (IGRT), and stereotactic body radiotherapy (SBRT) are examples of these advancements and allow increasing of the accuracy of dose delivery. This improves delivery precision, broadening the therapeutic index, and potentially reduces the side effects associated with treatment [1]. When treating cancer with RT, another critical challenge is represented by tumor radioresistance. Hence, the balance between radioresistance and radiosensitivity is of primary importance when choosing RT as a tool for cancer treatment [2]. As frequently observed, tumors differentially react to similar RT regimens. A prototypical example of differential radioresistance is given by breast cancers (BCs), a class of tumors that display high heterogeneity despite their same-tissue origin. In fact, breast cancer includes 21 distinct histological subtypes, and gene expression profiling has identified at least four molecular subtypes: luminal A, luminal B, HER2-enriched, and basal [3]. Recently, a fifth histotype has been identified, thus complementing the four previously established subtypes based on molecular profiling: triple-negative breast cancer (TNBC), which accounts for 10–15% of all BCs and represents one of the most aggressive and radioresistant forms [4]. While the classification of tumors by molecular profiling can be exploited to establish tumor-tailored therapies when considering chemotherapy and immunotherapy, the concept of personalized medicine is not applied to RT, considering it as a “one size fits all” type of treatment and thus neglecting the intrinsic and different radioresistances of breast neoplasms.

To propose a more personalized approach for RT, we recently developed and improved the local disease-free survival (LSR) model (Equation (1)). LSR aims to extend the LQ model, considering, together with the radiobiological parameters α and β, two other biological parameters, which are intrinsically cell-type dependent. One parameter is k, the number of clonogens; the second is Td, the average time of duplication of the cell line [5]:

LSR(D) = e^ −k e^(−αD − βD2 + ɣT) (1)

Dose regimens for BC vary, with hypofractionated and ultra-hypofractionated schedules having been demonstrated to be as effective and safe as conventional fractionation [6,7,8]. Due to the unique biological characteristics of TNBC as well as its clinical impact, ultra-hypofractionation is becoming a valid therapy option. Because of its aggressive nature, high risk of early recurrence, and lack of systemic treatment alternatives, TNBC necessitates prompt and efficient local therapy. Even though TNBC is very proliferative from a radiobiological standpoint (usually linked to a high α/β ratio), this does not necessarily support hypofractionation. Nonetheless, TNBC frequently displays flaws in DNA damage-repair mechanisms, which can make the patient more sensitive to radiation, especially when using high dose-per-fraction regimens. When taken as a whole, these traits support the idea of investigating ultra-hypofractionated regimens in TNBC [9,10,11]. To further implement the LSR model, we recently analyzed TNBC cell line BT549 together with MDA-MB-231. Both cell lines are aggressive and have a high metastatic potential; the first shows a more epithelial morphology, while the latter has a more mesenchymal phenotype. While MDA-MB-231 represents one of the most characterized cell lines in our previous studies, the BT549 cell line was chosen to add a less-featured model in radiobiological investigations. The comparison between the two cell lines was, furthermore, aimed at mimicking the biological variability in BC.

The goal of this work was to define ultra-hypofractionated treatment plan schedules exploiting the LSR with a biological approach in order to propose personalized RT strategies for TNBC.

## 2. Materials and Methods

### 2.1. Cell Cultures and Radiation Treatments

The human TNBC MDA-MB-231 cell line was purchased from the American Type Culture Collection (ATCC, Manassas, VA, USA) and cultured according to standard conditions, as previously described [9]. The human TNBC BT-549 cell line was kindly provided by Prof. Yary Ciribilli (University of Trento, Italy) and grown in RPMI-1640 supplemented with 10% FBS and 1% Insulin–Transferrin–Selenium (Gibco-Thermo Fisher Scientific, Waltham, MA, USA).

Cell irradiations were carried out with photon beams of 6 MV by using a medical Linac accelerator (Siemens Medical Systems, Concord, CA, USA) and a dose range from 1 to 5 Gy, as previously described [5].

### 2.2. Clonogenic Assay, Dose–Response Curves, and α and β Parameter Determination

Radiobiological characterization of the MDA-MB-231 and BT-549 cell lines was performed, and the surviving fraction (SF) values after cell irradiation were obtained by a clonogenic assay carried out as previously described [5,9]. Four replicates were used for each dose point and three independent irradiation experiments were performed. Dose–response curves were achieved by fitting the SF values to the linear–quadratic equation by using MATLAB software v.R2025a, and the (α) and (β) parameters were calculated as previously reported [5]. Since cell lines often display changes in their α/β ratios due to the heterogeneity in the cell cycle phases of the irradiated population, we conducted several irradiation experiments to evaluate the range of variations in these parameters [12].

### 2.3. Local Disease-Free Survival-Rate Model Application

The FAST FORWARD treatment scheme involves delivering 26 Gy in 5 fractions of 5.2 Gy each. A sequential tumor-bed radiotherapy boost to the conserved breast is possible [8]. Since TNBC is a key factor associated with poorer prognosis and is frequently linked to higher tumor grades, we considered a tumor-bed boost to be a mandatory component of our evaluation [13]. The LSR value was calculated using Equation (1) (please note that the term γT could be very small or negligible) for the medians, maximums, and minimums of the alpha and beta parameters of both cell lines, including the sequential boost on or off the tumor bed, to reach an LSR probability of close to 100%. For the MDA-MB-231 cell line, td was 25.39 and k was 47.39, while for the BT549 cell line, td was 33 and k was 48.

### 2.4. Treatment-Planning System Simulation

Based on the results obtained from the application of the LSR model, fifteen treatment plans of BC patients previously treated with deep-inspiration breath-holding were generated to simulate the new scenario. Each plan was optimized to ensure comprehensive coverage of the whole-breast clinical target volume, incorporating a 7 mm margin to define the planning target volume. A 2-arc volumetric modulated-arc therapy technique was employed, utilizing 6 MV to flatten filter-free photon beams on a Linac platform to enhance dose delivery efficiency. Dose constraints for the organs at risk (OARs) were applied following the FAST FORWARD protocol with the goal of maintaining an optimal balance between target coverage and OAR sparing, ensuring both treatment effectiveness and patient safety.

## 3. Results

### 3.1. Dose–Response Curves and (α) and (β) Parameter Determination

**Figure 1:**
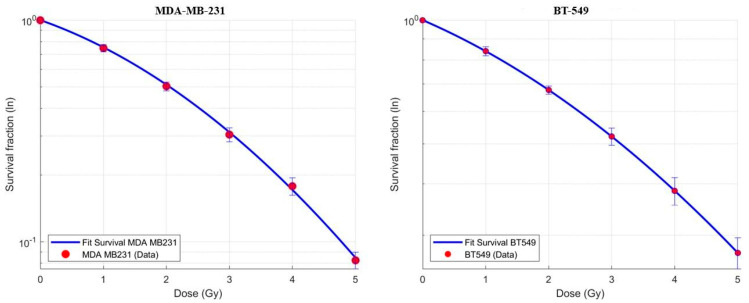
Dose–response curves.

The MDA-MB-231 and BT-549 TNBC cell lines were irradiated with increasing doses from 1 to 5 Gy of X-rays by using a medical linear accelerator. After performing of the clonogenic assay, the surviving fraction (SF) values were plotted to obtain dose–response curves according to the LQ model. The two dose–response curves are shown in Figure 1.

We identified three different α/β ratio values obtained in different experimental sessions: the minimum, the maximum, and the median ones.

The median value was preferred to the mean one because the median is less sensitive to extreme values (outliers) compared to the mean. Specifically, for MDA-MB-231, the α/β ratios were 3.79 Gy as the minimum, 15 Gy as the maximum, and 7 Gy as the median. For BT-549, the α/β ratios were 5.95 Gy as the minimum, 22.93 Gy as the maximum and 16.51 as the median.

### 3.2. Application of the Local Disease-Free Survival-Rate Model

The results of the FAST FORWARD scheme with and without the sequential boost are shown in Table 1, where it can be observed that most treatment regimens are not 100% effective.

To obtain a LSR probability of close to 100% for both cell lines according to the α/β ratios, the doses for fractions and boosts were optimized and are reported in Table 2. On the contrary, in this case, the treatment regimens are all 100% effective.

### 3.3. Treatment-Planning System Simulations

Among the different treatment schedules calculated, we selected the one with the highest dose per fraction, 9.9 Gy × 5, to simulate the worst-case scenario. Most plans failed to meet the dosimetric objectives while also not achieving full target coverage. The FAST FORWARD protocol requires the following planning target volume (PTV) dose distribution: more than 95% of the PTV must receive 95% of the prescribed dose, less than 5% of the PTV may receive 105% or more, less than 2% of the PTV may receive 107% or more, and the global maximum must remain below 110%. In the new plans, clinical target volume (CTV) coverage ranged from 75% to 90% of the prescribed dose, while PTV coverage ranged from 70% to 82% of the volume, receiving 95% of the prescribed dose. Maximum doses of 105% and 107% always remained within protocol thresholds. The mean (±standard deviation) CTV dose was 49.3 ± 0.3 Gy, slightly below the prescribed dose. Dose constraints for the five-fraction schedules were as follows: the volume of the ipsilateral lung receiving 8 Gy had to be less than 15%, while the volume of the heart receiving 1.5 Gy had to be less than 30% and the volume of the heart receiving 7 Gy had to be less than 5%. The median ipsilateral lung volume receiving 8 Gy was 21%, with values ranging from 17% to 27%. Similarly, the median heart volume receiving 1.5 Gy was 33%, with values ranging from 29% to 40%. However, the heart volume receiving 7 Gy always remained below the 5% threshold, with a median value of 4% and a range from 2% to 5%. The mean dose to the contralateral breast ranged from 1.9 Gy to 3.2 Gy, with a median dose of 2.6 Gy.

## 4. Discussion

TNBCs are characterized by high tumor cell proliferation, intrinsic radioresistance, and, consequently, poorer prognosis [13]. On these bases, medical research has focused on the identification of novel anticancer agents to improve the TNBC therapeutic landscape. In particular, an interesting class of antitumor drugs is represented by immune checkpoint inhibitors (ICIs). ICIs work by blocking proteins called checkpoints, which normally prevent the immune system from attacking cancer cells. For example, the ICI Pembrolizumab has demonstrated remarkable efficacy in clinical trials, leading to its integration into the TNBC treatment paradigm [14,15,16]. Accordingly, the KEYNOTE-522 phase III trial [17] evaluated 602 TNBC patients who received standard neoadjuvant chemotherapy combined with either Pembrolizumab or a placebo. That study reported a significant increase in the rate of pathological complete responses (pCRs) when immunotherapy was added to neoadjuvant chemotherapy. Based on this strong evidence, international guidelines now recommend the combination of Pembrolizumab and chemotherapy as the new standard of care for early-stage TNBC [18].

In this rapidly evolving therapeutic scenario, it is crucial to also redefine the role of adjuvant RT, which remains a cornerstone in TNBC management. Our findings suggest that dose escalation plays a pivotal role in achieving a higher rate of tumor cell eradication. Optimizing RT strategies, particularly in the context of emerging systemic therapies, is essential to improving disease control and long-term outcomes in TNBC patients. As for the treatment of tumors with chemotherapy, there is a growing need to develop personalized RT approaches to optimize treatment efficacy and minimize toxicity. Personalized RT strategies could enhance therapeutic outcomes, particularly in radioresistant tumors where RT plans should be designed based more on the biological characteristics of the tumors to be treated [9].

As a proof of concept, we recently developed a generalized LQ model in vitro to calculate the LSR, taking into account, as novelty, the doubling time (Td) and the number of clonogens (k), exclusive to each BC cell line, in addition to the α/β ratio, which may vary within the same cell line [5]. In the present study, we applied the LSR model to two different TNBC cell lines, MDA-MB-231 and BT-549, in order to address personalized RT plans in terms of dose per fraction and total dose for both cell lines and to reach an LSR probability of close to 100%. While MDA-MB-231 represents one the most characterized cell lines both from the literature and from our previous studies, the BT549 cell line was added, being a less-featured TNBC model in radiobiological investigations. However, to overcome the limitations coming from the study of only two cell lines and to mirror the heterogeneity of TNBCs, further in vitro experiments are currently under investigation to enlarge and test our results to other TNBC cell lines as well. Of course, in vitro data should be considered as preliminary ones before testing our experimental evidence in vivo. This will allow us not only to validate the efficacy of the results obtained in vitro but also to translate them into a more complex and reliable setting. Moreover, in vivo experiments will give us more insights on the side effects generated by the application of our model.

Integrating LSR modeling into the clinical RT management of TNBC could pave the way for more effective, individualized RT radiotherapy protocols, ultimately based on biological factors rather than conventional treatment guidelines. The variation in α/β values observed across the experimental sessions highlights the complexity of TNBC’s response to radiation. Given TNBC’s known heterogeneity, our results align with previous studies suggesting that a one-size-fits-all RT approach may not be effective for all patients.

Unfortunately, studies proving, in vitro, the efficacy of hypofractionation against normal RT regimens are very few. For example, Groshe et al. compared 2 Gy per fraction vs 2.67 Gy per fraction, demonstrating equal effectiveness in terms of cell viability and toxicity in TNBC cell lines [11].

Another relevant implication of our findings is the potential integration of the LSR model into clinical treatment-planning software. Current RT planning largely relies on generic dose constraints and does not account for individualized tumor radiosensitivity. Incorporating LSR-based predictions could enable radiation oncologists to personalize treatment regimens more effectively, potentially improving success rates while minimizing toxicity to surrounding healthy tissues.

However, our TPS analysis demonstrated that the dose regimens suggested by the LSR model might not be applicable in real-life clinical practice. Even the use of a breath-holding technique could not sufficiently mitigate the dosimetric impact of these schedules, which were found to exceed all dose constraints for OARs. Moreover, it is well-known that larger volumes receiving 107% or 110% of the prescribed dose are associated with an increased risk of late breast toxicity, particularly fibrosis [19,20]. To prescribe and administer 9.9 Gy × 5 fractions (190% of the 26 Gy) to the whole breast, while effective in vitro, would almost certainly cause unacceptable fibrosis in real patients.

This finding makes a dose-escalated regimen, based on the LSR of in vitro TNBC cells, inapplicable in standard clinical settings. However, these doses could be highly relevant in two specific scenarios: (1) as a tumor-bed boost during adjuvant radiation therapy and (2) in the treatment of oligometastatic patients with SBRT.

It is well-established that 90–95% of tumor recurrences occur within the original tumor bed, typically within 15 mm of the primary tumor site [21,22]. Bartelink et al. [23] demonstrated that in patients with poor prognostic features—such as younger age, grade 3 tumors, and close surgical margins—the administration of a 16 Gy boost in eight fractions significantly improved 10-year local control. In the context of dose escalation, Coles et al. [24], in a phase III randomized trial, found that a dose-escalated approach did not lead to improved disease control compared to the standard regimen. However, Livi et al. [25] retrospectively analyzed a large cohort of 2093 patients treated with adjuvant radiotherapy, including those who received boosts. After a median follow-up of 5.2 years, they identified triple-negative breast cancer (TNBC) as an independent predictor of poorer local control (*p* = 0.036). These findings suggest that dose escalation may be particularly beneficial in this subgroup of patients, and our results provide further support in this direction.

A dose-escalated approach may be even more suitable in the pre-surgical setting. Neoadjuvant radiotherapy has the inherent advantage of targeting well-defined and visible tumors, allowing for precise dose delivery in a smaller treatment volume while minimizing exposure to surrounding healthy tissues and uninvolved breast areas [26]. Delivering higher radiation doses to a limited target volume in this context could potentially achieve complete tumor eradication while maintaining adherence to organ-at-risk (OAR) constraints. In the neoadjuvant setting, it is well-established that achieving a pathological complete response (pCR) is associated with improved disease control [27]. However, data specifically evaluating neoadjuvant radiotherapy in triple-negative breast cancer (TNBC) remain scarce. Soares et al. [28] reported on a small cohort of 16 TNBC patients treated with preoperative radiotherapy, none of whom achieved a pCR. Our findings may contribute to optimizing radiotherapy protocols to increase the likelihood of achieving pCRs in TNBC patients while ensuring that the lowest possible dose is delivered to surrounding healthy structures.

Finally, in the oligometastatic setting, SBRT has been shown to have a favorable toxicity profile, with very low rates of adverse effects [29]. However, despite its safety, the impact of SBRT on disease outcomes in breast cancer remains uncertain. In this context, the NRG-BR002 trial, a randomized study investigating SBRT to all sites of metastases versus the standard of care (SOC) in oligometastatic breast cancer, did not demonstrate a significant survival benefit [30]. That study included 125 patients (SBRT = 65, SOC = 60), with a median follow-up of 30 months. The median progression-free survival was 23 months in the SOC group and 19.5 months in the SBRT arm, with no statistically significant difference. Similarly, the 3-year overall survival was comparable between groups (71.8% vs. 68.9%, *p* = 0.54).

Comparable results were observed in the CURB trial [31], a phase 2, open-label, randomized controlled trial evaluating SBRT in oligoprogressive breast cancer. The impact of SBRT was not significant compared to the SOC, with a median progression-free survival of 4.4 months versus 4.2 months (HR = 0.78; *p* = 0.43).

Given the challenges in achieving disease control, future strategies may focus on individualized, biologically driven dose prescription [32,33]. A potential future approach could involve tailoring SBRT indications based on the LSR of tumor cells directly obtained from metastatic biopsy sites. This could enable a fully personalized treatment strategy, optimizing disease control while minimizing toxicity.

Important considerations should be made regarding the applicability of our model in the modern era of artificial intelligence (AI), which is rapidly transforming the landscape of medicine. In the field of radiation oncology, AI represents a powerful tool for optimizing and automating dose-prediction and treatment-planning processes [34]. Our findings are not intended to serve as an alternative to or replacement for future AI-driven approaches. On the contrary, they provide valuable data that can serve as a foundation for algorithm training and validation—enabling the development of personalized dose schedules tailored to the characteristics of each patient and tumor. Nonetheless, additional data are urgently needed to strengthen and refine these models.

## 5. Conclusions

**Figure 2:**
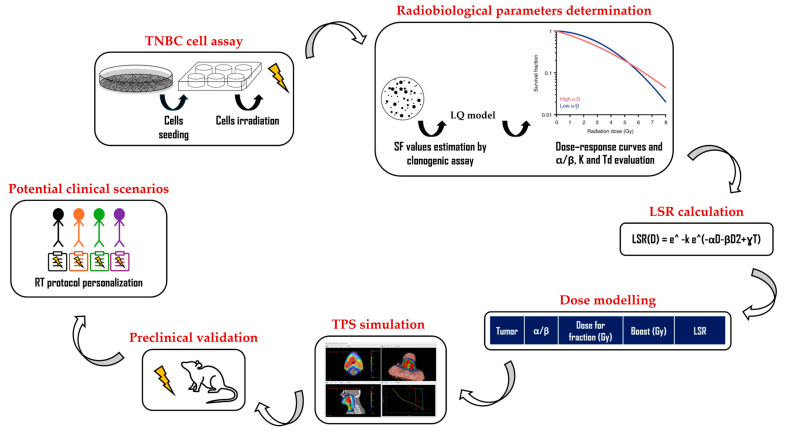
The multistep, biologically driven approach for the LSR model application.

This study supports the feasibility of a biologically driven RT approach (Figure 2) for TNBC, demonstrating that personalized ultra-hypofractionated regimens can optimize local disease-free survival. In the setting of adjuvant whole-breast irradiation, the doses predicted by the LSR model to achieve 100% cell death effectiveness in TNBC cannot be routinely delivered in the current clinical practice. Notably, a biologically driven approach to dose escalation in TNBC could be beneficial in the oligometastatic or neoadjuvant setting, where higher doses can be easily applied on limited volumes.

In order to evaluate our findings, we will plan to test them in vivo using a mouse model of TNBC. Such a step will be of pivotal importance to test the effectiveness and underline potential negative effects of our proof of concept. In addition, further research is needed to validate these findings and explore their broader applicability in other cancer types.

## Figures and Tables

**Table 1:** LSR for FAST FORWARD with or without boost administration.

Cell Line	Alpha/Beta	Dose for Fraction (Gy)	Boost (Gy)	LSR
BT549	16.51	5.2	-	82.0%
BT549	5.95	5.2	-	84.0%
BT549	22.93	5.2	-	75.0%
BT549	16.51	5.2	2	96.5%
BT549	5.95	5.2	2	97.4%
BT549	22.93	5.2	2	94.6%
MDA	7	5.2	-	100%
MDA	3.79	5.2	-	93.7%
MDA	15	5.2	-	99.9%
MDA	7	5.2	2	100%
MDA	3.79	5.2	2	98.5%
MDA	15	5.2	2	100%

**Table 2:** New dose schedules to obtain an LSR of 100%.

Cell Line	Alpha/Beta	Dose for Fraction (Gy)	Boost (Gy)	LSR
BT549	16.51	9.1	-	100%
BT549	5.95	8	-	100%
BT549	22.93	9.9	-	100%
BT549	16.51	5.2	5.6	100%
BT549	5.95	5.2	5	100%
BT549	22.93	5.2	6.2	100%
MDA	7	5.2	-	100%
MDA	3.79	7.3	-	100%
MDA	15	5.3	-	100%
MDA	7	5.2	2	100%
MDA	3.79	5.2	4.4	100%
MDA	15	5.2	2	100%

## Data Availability

The datasets used and/or analyzed during the current study are available from the corresponding author on reasonable request.

## Abbreviations

The following abbreviations are used in this manuscript:

TNBC	Triple-negative breast cancer
LSR	Local disease-free survival
RT	Radiotherapy
IMRT	Intensity-modulated radiation therapy
IGRT	Image-guided radiation therapy
SBRT	Stereotactic body radiation therapy
SF	Surviving fraction
PTV	Planning target volume
CTV	Clinical target volume
pCR	Pathological complete response
SOC	Standard of care
OAR	Organ at risk
